# Comparison of the Analgesic Effect of Sufentanil versus Fentanyl in Intravenous Patient-Controlled Analgesia after Total Laparoscopic Hysterectomy: A Randomized, Double-blind, Prospective Study

**DOI:** 10.7150/ijms.34656

**Published:** 2019-09-20

**Authors:** Seok Kyeong Oh, Il Ok Lee, Byung Gun Lim, Hyerim Jeong, Young Sung Kim, Sul Gi Ji, Jong Sun Park

**Affiliations:** 1 Department of Anesthesiology and Pain Medicine, Korea University Guro Hospital, Korea University College of Medicine, Seoul, Republic of Korea.

**Keywords:** Patient-controlled analgesia, Fentanyl, Sufentanil, Postoperative pain, Hysterectomy.

## Abstract

**Background:** Fentanyl is one of the most widely used opioids for intravenous patient-controlled analgesia (IV-PCA). Sufentanil, a fentanyl analog, is suitable for postoperative pain control because it has no active metabolites and shows a higher therapeutic index and lower frequency of respiratory suppression than fentanyl. This study aimed to compare the two opioids for postoperative pain relief on the basis of analgesic efficacy, adverse effects, and patient satisfaction.

**Methods:** Sixty-four patients undergoing total laparoscopic hysterectomy were randomly allocated into a fentanyl group (n = 31) or a sufentanil group (n = 33). The patients received 50-μg fentanyl or 10-μg sufentanil before induction of anesthesia and 5 minutes after uterine incision during surgery in the fentanyl and sufentanil group, respectively. After arriving at the post-anesthesia care unit (PACU), verbal pain score (VPS) and sedation score were assessed. IV-PCA (fentanyl 1250 μg or sufentanil 250 μg with ondansetron 8 mg; total volume, 60 ml) was connected and continued for 48 h postoperatively. Postoperative pain was evaluated by using the numeric rating scale (NRS; at rest/during cough) at 6, 12, 24, 36, and 48 hours after surgery. The cumulative PCA consumption, patient satisfaction scores, and adverse effects were measured.

**Results:** In the PACU, VPS was significantly higher and rescue fentanyl consumption was higher in the fentanyl group than in the sufentanil group, while the sedation score and adverse effects were comparable between the groups. No significant differences were observed in the NRS scores for pain (at rest/during cough) in the ward over 48 hours postoperatively, but the cumulative PCA consumption was significantly higher in the fentanyl group (47.4 ± 9.9 ml vs. 36.2 ± 14.6 ml, *P* = 0.01). There were no significant intergroup differences in patient satisfaction score and the incidence of adverse effects in the ward, except for a higher incidence of dry mouth in the fentanyl group.

**Conclusions:** In comparison with fentanyl, sufentanil showed comparable analgesic efficacy and safety with less analgesic consumption (under a potency ratio of 1:5) in IV-PCA after total laparoscopic hysterectomy. Therefore, we suggest that sufentanil can be a useful alternative to fentanyl for IV-PCA.

## Introduction

Intravenous patient-controlled analgesia (IV-PCA) is frequently used to relieve postoperative pain in hospitalized patients by titrating analgesics on demand 1. Opioids are typically used for IV-PCA, and fentanyl is one of the most frequently used opioids for this purpose 2 because of its enhanced analgesic efficacy and potency and fewer adverse effects than morphine or meperidine 3. As a fentanyl analog, sufentanil is a highly lipophilic synthetic piperidine derivative opioid that has high affinity for μ-opioid receptors and is suitable for postoperative pain control because it has no active metabolites and shows a higher therapeutic index and lower frequency of respiratory suppression than fentanyl 4. Nevertheless, there have been few studies 5, 6 comparing fentanyl and sufentanil with respect to their analgesic efficacy for IV-PCA.

Total laparoscopic hysterectomy (TLH) is a commonly performed procedure in gynecological practice and offers advantages such as rapid recovery, less postoperative morbidity, reduced postoperative pain, and shorter hospital stay, in comparison with abdominal hysterectomy 7. Despite these advantages, TLH is often followed by an unexpectedly high level of pain; because the pain after TLH is underestimated in comparison with that after abdominal hysterectomy, pain control is often inadequate, with comparably lower doses of opioid used 8. Uncontrolled pain after surgery can reduce patient satisfaction, cause chronic postoperative pain development 9, and increase cardio-pulmonary complications and morbidity and mortality 10-12.

This study, therefore, was performed to compare the postoperative pain relief afforded by fentanyl and sufentanil in terms of their analgesic efficacies, including pain scores and opioid consumption, adverse effects, and overall satisfaction, in patients undergoing TLH. We hypothesized that sufentanil could provide comparable analgesic efficacy with lower postoperative respiratory depression than fentanyl in IV-PCA after TLH.

## Methods

### Patients and study design

This prospective double-blinded randomized study was approved by the Korea University Guro Hospital Institutional Review Board, Seoul, Republic of Korea (KUGH16346-001), and by the Ministry of Food and Drug Safety (MFDS, Korea), formerly known as the Korea Food and Drug Administration (KFDA), on December 23, 2016 (clinical trial approval number: 31136), and was conducted from April 2017 to January 2018 as a single-center trial. Written informed consent was obtained from every patient who was scheduled to undergo elective TLH under general anesthesia, was a female aged 19-75 years, and had an American Society of Anesthesiologists physical status I-II. Patients were excluded if they had body mass index > 30.0 kg/m^2^, known hypersensitivity to the drugs used in this study, significant liver or renal dysfunction, or a history of drug abuse or dependence, recent major procedure or surgery, or preoperative analgesic use.

Participants were randomly assigned to either a fentanyl or a sufentanil group by a web-based computer-generated list and were unaware of their assignment. The randomized numbers were kept by the qualified clinical research pharmacist who managed the drugs; the drugs were delivered in a sealed opaque envelope before surgery; and the envelope was opened in the operating room only by a non-blinded anesthesiologist who was responsible for anesthesia management and setting the PCA pump as per the protocol in the operating room. Other investigators who assessed the study endpoints after the operation in the post-anesthesia care unit (PACU) and the ward were blinded to the group assignment. All the PCA devices were applied to patients with labels with only the patient's study number, so that neither the patients nor medical care providers and investigators could recognize the PCA regimen.

### Anesthesia and PCA regimen

All patients were premedicated with intramuscular midazolam 2 mg and glycopyrrolate 0.2 mg 30 minutes before anesthesia. In the operating room, all patients underwent routine physiological monitoring, which included pulse oximetry, electrocardiography, and noninvasive arterial blood pressure measurements. Bispectral index (BIS; BIS VISTA™; Aspect Medical Systems Inc., Norwood, MA, USA) was determined to monitor the depth of hypnosis, and maintained from 40 to 60 during surgery.

For preemptive pain control and maintenance of hemodynamic stability during the initial phase of the operation, fentanyl 50 μg in the fentanyl group and sufentanil 10 μg in the sufentanil group were administered intravenously immediately before induction of anesthesia. Anesthesia was induced with intravenous administration of propofol 2 mg/kg, followed by rocuronium 0.6 mg/kg, which was administered after loss of consciousness (BIS < 60) to facilitate endotracheal intubation, and maintained with desflurane and 50% nitrous oxide in oxygen. The concentration (vol%) of desflurane was guided by the BIS value. The core temperature was maintained at approximately 36°C using a warm air blower (3M™ Bair Hugger™ Intraoperative Blankets, 3M™, St. Paul, MN, USA). Five minutes after uterus incision, fentanyl 50 μg in the fentanyl group and sufentanil 10 μg in the sufentanil group was administered to control the pain from surgical stimulus during surgery. At the end of the surgical procedure, neuromuscular blockade was reversed with glycopyrrolate 0.4 mg and pyridostigmine 10 mg. Tracheal extubation was performed after confirming the response to verbal commands and spontaneous respiration. If mean arterial pressure decreased or increased by more than 30% from the baseline value, ephedrine 4 mg or nicardipine 0.5 mg, respectively, was administered.

In the PCA protocol, the dose was based on a previous review article 13 and a recent study by Kim et al. 5 In the fentanyl group, IV-PCA was performed with fentanyl 1250 μg and ondansetron 8 mg mixed with 0.9% isotonic saline to a total volume of 60 ml, whereas the patients in the sufentanil group received IV-PCA with sufentanil 250 μg and ondansetron 8 mg mixed with 0.9% isotonic saline to a total volume of 60 ml. The PCA device used in the study was Anaplus® AP0605 (E-WHA biomedics, Seoul, Republic of Korea) as an elastomeric pump, with a basal rate of 0.5 ml/h, and a lockout period of 15 minutes, and a single bolus injection volume of 0.5 ml and maximal volume of 60 ml; thus, the maximal limit per hour was 2.5 ml. Through this PCA device, the fentanyl group received a basal infusion of 10.4 μg/h with a bolus dose of 10.4 μg over 15 minutes and the sufentanil group received a 2.08-μg/h basal infusion with a bolus dose of 2.08 μg over 15 minutes.

### Postoperative recovery and pain management

After arrival at the PACU, the patient's pain level was evaluated according to a verbal pain score (VPS; 0-3; 0 = no pain, 1 = mild pain, 2 = moderate pain, 3 = intense pain) 14 at 10-minute intervals, and the sedation score (0-3; 0 = clearly conscious; 1 = temporarily drowsy; 2 = drowsy but responsive to verbal communication; and 3 = drowsy without response to verbal communication) 14 was also assessed at 10-minute intervals. Fentanyl 20 µg was administered if the patient showed VPS ≥ 2, respiratory rate ≥ 10 per minutes, SpO_2_ ≥ 95%, and sedation score ≤ 1. When the VPS and the sedation score were ≤1, the PCA device was connected and PCA was commenced. If the VPS was not ≤1 despite repetitive rescue injections of fentanyl 20 µg at 10-minute intervals over 2 hours, the case was considered to represent failure in pain control and excluded from the analysis group.

After the patients were transferred to the ward, and the pain level was evaluated according to a numeric rating scale (NRS; 0-100; no pain [0] to worst pain imaginable [100]) at 6, 12, 24, 36, 48 hours after surgery. The cumulative consumption of PCA over 48 hours and the occurrence of adverse effects (e.g., nausea and vomiting, dry mouth, dizziness, urinary retention, headache, sedation, itchiness, shivering, respiratory depression, confusion, hypotension, and bradycardia) were assessed. Respiratory depression was defined by respiratory rate < 10 per min or oxygen saturation < 90% for >1 min 15. The patient's overall satisfaction score (0-3; 0 = un-satisfied, 1 = partial-satisfied, 2 = satisfied, 3 = full-satisfied) was also evaluated. If the postoperative pain control in the ward was insufficient (NRS score for pain > 30), 50 µg of fentanyl as a rescue analgesic was planned to be administered. The duration of action of intravenous fentanyl is known to be 30 to 60 minutes; therefore, the rescue dose was not administered at least 1 h before the evaluation time points; but if it was inevitable, the evaluation time point was delayed by 1 h after the administration.

### Evaluation of outcomes

The outcomes measured included (1) VPS (0-3) measured every 10 minutes at the PACU; (2) the sedation score (0-3) measured every 10 minutes at the PACU; (3) rescue fentanyl administration in the PACU; (4) PCA connection time at the PACU (min); (5) NRS score (0-100) at rest and during cough in the ward (primary endpoint); (6) patient's overall satisfaction score (0-3); (7) cumulative PCA consumption (ml); (8) occurrence of side effects in the PACU and in the ward for 48 hours after PCA connection.

### Statistical analysis

A pilot study prior to the study was impossible because the use of sufentanil for PCA in Korea was not approved by the MFDS, Korea, formerly known as the KFDA. However, based on a previous similar study 5 that examined 42 patients per group, we calculated the sample size expecting to obtain similar results in our study. If the lower boundary for the difference in the pain score at 24 hours after surgery was not less than -10%, sufentanil treatment would be considered non-inferior to fentanyl treatment. On the basis of the assumption that the allocation ratio of the two groups was 1, a sample size of 29 patients was selected for each group, calculated by a non-inferiority test with a significance level of 0.05, power of 0.9, and non-inferiority margin of 10%. We aimed to assign 35 patients to each group after accounting for 15% drop-outs.

Statistical analyses were performed using the SPSS software (version 20.0; IBM, USA). The normal distribution of continuous data was first evaluated using the Shapiro-Wilk test (*P* >0.05). The normally distributed data were analyzed using Student's t-test, and the abnormally distributed data were analyzed using Mann-Whitney U test. Student's t-test was used to compare the age, height, weight, anesthesia time, operation time; while the Mann-Whitney *U* test was used for rescue fentanyl dose and PCA connection time at the PACU, the NRS score at rest and during cough at 6, 12, 24, 36, 48 hours after surgery, and the cumulative PCA consumption.

Ordinal parameters, including the VPS and sedation score at the PACU and patient's overall satisfaction score, were compared using the Mann-Whitney U test, while categorical variables, including the American Society of Anesthesiologists (ASA) classification and the incidence of adverse events, were compared by a chi-squared test or Fisher's exact test. The changes over time in the VPS and sedation score at the PACU, the NRS score for pain, and cumulative PCA consumption in the ward were compared using repeated-measures analysis of variance.

Data are expressed as the mean ± SD or number of patients (%). *P* values were two-tailed, and a *P* value of less than 0.05 was considered to be statistically significant.

## Results

**Figure 1:**
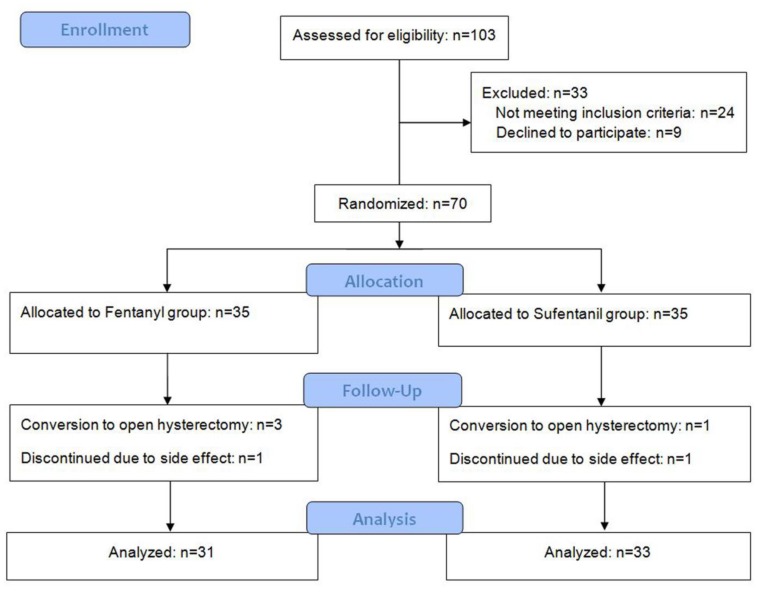
A flowchart describing patient recruitment, randomization, and withdrawal.

A total of 103 patients were assessed for eligibility, and 33 patients were excluded for noncompliance with the study protocol; thus, 70 patients (35 for each group) were randomized. Among these, four operations were converted to open hysterectomy and two patients discontinued IV-PCA due to adverse effect (severe nausea). Finally, 31 patients in the fentanyl group and 33 in the sufentanil group were analyzed (Figure 1). There was no significant intergroup difference in baseline patient characteristics and operation and anesthesia times (Table 1).

**Figure 2:**
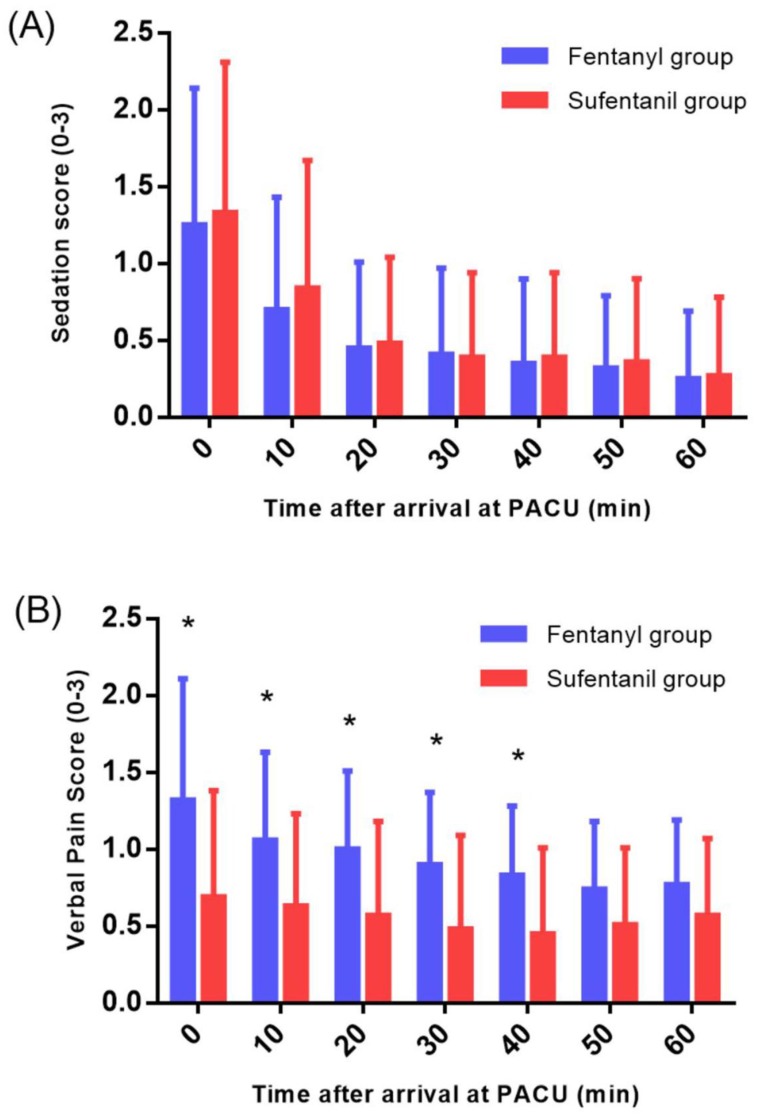
Sedation score (A) and verbal pain score (B) at the PACU. PACU: post-anesthesia care unit. Sedation score (0 = clearly conscious; 1 = temporarily drowsy; 2 = drowsy but responsive to verbal communication; and 3 = drowsy without response to verbal communication). Verbal pain score (0 = no pain, 1 = mild pain, 2 = moderate pain, and 3 = intense pain). ^*^P < 0.05.

At the PACU, the fentanyl group showed significantly higher VPS (*P* = 0.001) (Figure 2) and higher rescue fentanyl administration (22.1 ± 26.1 vs. 8.3 ± 17.3; *P* = 0.005) than the sufentanil group, while the sedation score and adverse effects showed no intergroup differences (Table 2).

**Figure 3:**
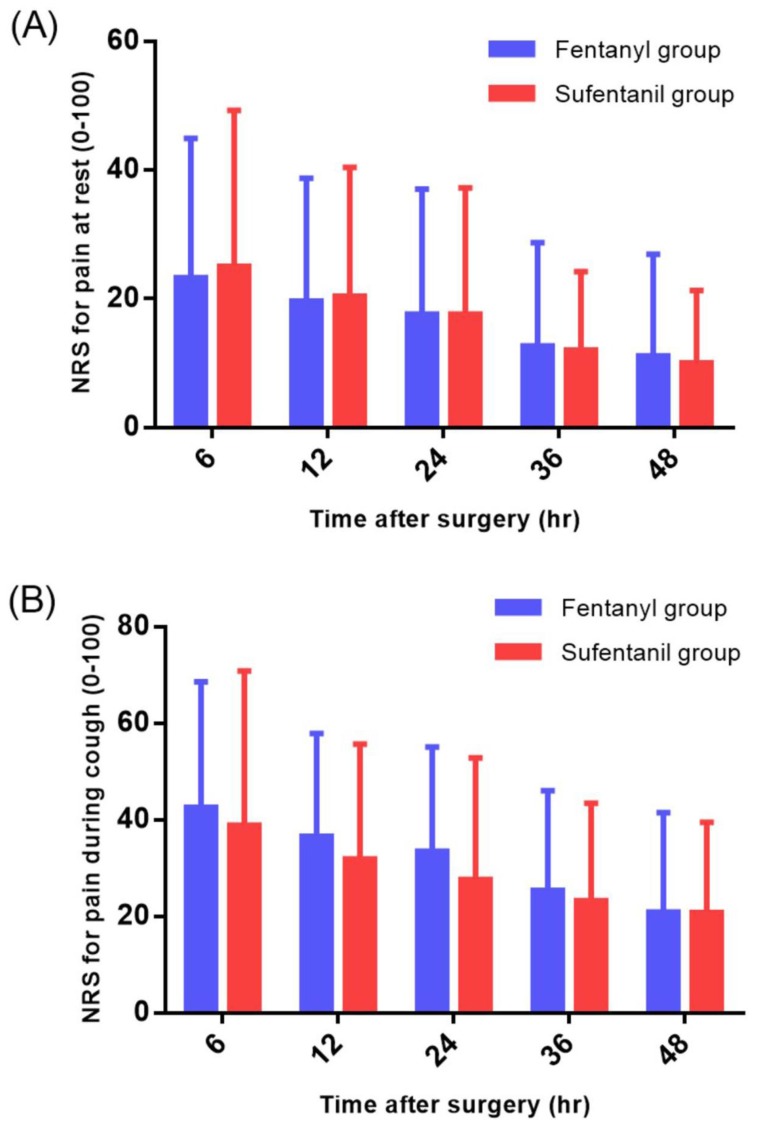
Numerical rating scale (NRS) for pain at rest (A) and during cough (B) in the ward.

**Figure 4:**
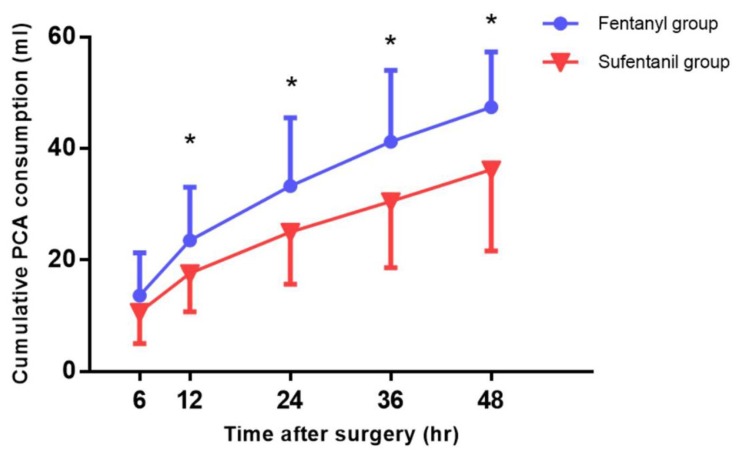
Cumulative patient-controlled analgesia (PCA) consumption in the ward. *P < 0.05.

No significant differences were observed in the NRS scores for pain at rest and during cough in the ward during the 48-hour postoperative period (Figure 3), but the cumulative consumption of PCA during this period in the ward was significantly higher in the fentanyl group than in the sufentanil group (47.4 ± 9.9 ml vs. 36.2 ± 14.6 ml,* P* = 0.01) (Figure 4).

## Discussion

In the present study, both fentanyl and sufentanil IV-PCA provided satisfactory postoperative pain control. Immediately after surgery, at the PACU, patients who received sufentanil showed lower pain scores and rescue analgesic requirement while the sedation score and incidence of adverse effects were not different between the two groups. In addition, over 48 hours after surgery, patients who received sufentanil IV-PCA showed less cumulative consumption compared to those who received fentanyl IV-PCA, with comparable analgesic efficacy and adverse effects (except the lower incidence of dry mouth in the sufentanil group).

Opioids are useful and potent analgesics for relieving moderate-to-severe postoperative pain. Morphine, fentanyl, and sufentanil are some of the commonly used opioids for IV-PCA 2, 13, 16, 17. Morphine has been regarded as the first choice for IV-PCA and is the most commonly used and the most studied drug for IV-PCA 13. However, the usefulness of morphine is sometimes compromised by its active metabolite—morphine-6-glucuronide—that also produces respiratory depression, especially in patients with renal insufficiency because the metabolite is mainly excreted by the kidney 18. Fentanyl can be a good alternative for morphine-intolerant patients or those with altered renal function because it has no active metabolites and does not rely on renal excretion for elimination. Moreover, because of its lipophilicity, fentanyl has a quicker onset than morphine, possibly making it better suited for IV-PCA 13. Therefore, fentanyl has been successfully used and is one of the most frequently used opioids for IV-PCA, especially in Korea 2.

A fentanyl progenitor, sufentanil, shows some advantages over other opioids. Sufentanil, like fentanyl, has no active metabolites. It is 5-10 times more potent than its parent drug fentanyl 5, 19. The therapeutic index of sufentanil was markedly higher (26716) than those of morphine (71) and fentanyl (277) in preclinical models; this factor has clinical significance due to the lower incidence of respiratory depression with sufentanil than that with the other opioids 4, 16. Sufentanil has a relatively rapid equilibration half-life (t½ke0; 6.2 minutes) between plasma and brain, compared to 2.8 hours for morphine 20, 21, and a relatively shorter duration of action than morphine and fentanyl 22. Based on these properties, sufentanil can be considered to be appropriate and suited for IV-PCA. It is the most commonly used opioid for IV-PCA in China 17, 23, but there is relatively scarce evidence for its use in IV-PCA.

In our study, the sufentanil group consumed significantly less cumulative IV-PCA to maintain a pain score comparable with that in the fentanyl group. Mean cumulative consumption of PCA at 48 h after the operation in the sufentanil group was 36.2 ml, while that in the fentanyl group was 47.4 ml. Considering the weight of the participants, the PCA regimen was composed of about 4 µg/kg of sufentanil and 20 µg/kg of fentanyl in the respective groups, and during the postoperative 48-h period, 3.14 µg/h (0.0514 µg/kg/h) of sufentanil and 20.57 µg/h (0.3344 µg/kg^/^h) of fentanyl were respectively consumed. Given the comparable efficacy and adverse effects, the potency ratio was calculated as approximately 1:6.5 from the consumed dose. Therefore, the preset potency ratio of 1:5 in our study seemed to be somewhat greater in sufentanil than in fentanyl. In a previous study that compared fentanyl and sufentanil in IV-PCA after lumbar fusion 5, the potency ratio was 1:6 (4 µg/kg for sufentanil and 24 µg/kg for fentanyl), and sufentanil IV-PCA showed comparable analgesic efficacy and lower incidence of postoperative nausea and vomiting (PONV).

A basal infusion may be required in sufentanil IV-PCA because of the short half-life of clearance, otherwise patients will require frequent boluses. However, previous studies have cautioned against using basal infusions routinely in IV-PCA for opioid-naive patients 13, 24-26 because of the increased risk of respiratory depression associated with opioids. Nevertheless, as described above, sufentanil has the highest therapeutic index and a lower respiratory depressive property in comparison with other opioids; 4, 16, 26, 27 thus, the potential problems associated with the use of basal infusions can be minimized with sufentanil. The major safety issue related to the use of IV-PCA is respiratory depression, which can be potentially life-threatening although it is not common 13. The incidence of respiratory depression with IV-PCA has been reported to range from 0.19% to 5.2% 28. The variations in the incidence could be attributed to the different definitions of respiratory depression, which is usually defined as reduced respiratory rate under 8 or 10 breaths per minute, sometimes including its depth and rhythm, and/or with oxygen saturation under 85%-90% 28. Our data showed no respiratory depression associated with the use of both sufentanil and fentanyl (considering the definition of respiratory rate < 10 or oxygen saturation < 90% for >1 min) despite the basal infusion. This might be attributable to the relatively higher therapeutic index of fentanyl and sufentanil.

The commonly observed adverse effects of opioid-based PCA are nausea and vomiting, pruritis, urinary retention, sedation, and, less commonly, respiratory depression and confusion 13. Among these, PONV is the most common and most bothersome adverse effect of opioid-based IV-PCA, and the risk factors for PONV include female sex, a history of motion sickness or PONV, nonsmoking status, and postoperative opioid use 29. Our study population consisted of female patients who were receiving opioids postoperatively; therefore, for antiemetic prophylaxis, ondansetron 8 mg was administered. In previous studies comparing sufentanil and fentanyl 5, 6, the incidence of PONV was significantly lower in patients receiving sufentanil than in those receiving fentanyl. In contrast, our results showed no intergroup difference in the incidence of PONV and a higher incidence of moderate-to-severe PONV (39% in the ward) than in the previous two studies (4.8%-10%) when sufentanil used. In other studies, the incidence of PONV in sufentanil only IV-PCA was 31%-35% 30, 31, which was not much lower than our results. The higher incidence of PONV might be related to non-patient factors, including the type of surgical procedure (laparoscopy) and drugs used during anesthesia (inhalational agent than propofol), since it was reported that the incidence of PONV increased with the number of risk factors 32, 33.

Dry mouth (xerostomia) is an underestimated opioid-induced side effect. Normal salivation is very important for oral health because it contributes to oral defense mechanisms, and impaired saliva secretion may cause dental caries or mucosal deterioration 34. Opioids are well‐known causes of dry mouth 35, 36, although the mechanism underlying this effect is unclear. A sufentanil-based IV-PCA regimen can be chosen instead of a fentanyl-based regimen to attenuate the incidence of dry mouth, although more evidence is required to support this advantage of sufentanil.

This study has some limitations. First, the 1:5 potency ratio of sufentanil to fentanyl seemed to be slightly higher for sufentanil. This might have affected the superior results showing lower pain scores and rescue analgesic requirement at the PACU and less cumulative PCA consumption of sufentanil. However, considering the consumed dose for comparable efficacy, the ratio was estimated to 1:6.5, which can be considered as an equivalent dose ratio of fentanyl to sufentanil in IV-PCA. Second, the prophylaxis for PONV might have been insufficient. The risk factors for PONV in this study were female sex, postoperative opioid use, and laparoscopy; therefore, it would have been better to administer not only ondansetron (serotonin antagonist) but also other antiemetics (e.g., droperidol and dexamethasone) for combination therapy.

## Conclusions

In comparison with fentanyl, sufentanil showed comparable analgesic efficacy and safety (except the lower incidence of dry mouth) with less analgesic consumption under a potency ratio of 1:5 in IV-PCA over the 48-h period after TLH. Therefore, we suggest that sufentanil can be a useful alternative to fentanyl for IV-PCA.

## Figures and Tables

**Table 1:** Demographic and clinical data

	Fentanyl (n=31)	Sufentanil (n=33)	*P*-value
Age (year)	49.9 ± 8.0	49.6 ± 7.3	0.819
ASA (I/II)	11/20	13/20	0.749
Height (m)	1.56 ± 0.05	1.57 ± 0.06	0.280
Weight (kg)	61.0 ± 11.6	61.5 ± 8.1	0.844
Operation time	74.4 ± 32.8	72.1 ± 34.0	0.790
Anesthesia time	115.2 ± 32.7	109.9 ± 35.2	0.509

Values are mean ± SD or number of patients. ASA: American Society of Anesthesiologists physical status classification.

**Table 2:** Postoperative outcomes

	Fentanyl (n=31)	Sufentanil (n=33)	*P*-value
PACU outcomes			
Rescue Fentanyl (mg)	22.1 ± 26.1	8.3 ± 17.3	0.005
PCA connection time (min)	11.6 ± 9.6	8.1 ± 10.1	0.094
Nausea	5 (16.1)	6 (18.2)	1.000
Vomit	1 (3.2)	1 (3.0)	1.000
Adverse effects at ward			
Nausea (severe/moderate/mild/none)	3/4/4/20	7/6/6/14	0.334
Vomiting	2 (6.5)	1 (3.0)	0.607
Dry mouth	15 (48.4)	6 (18.2)	0.016
Dizziness	6 (19.4)	12 (36.4)	0.169
Urinary retention	7 (22.6)	4 (36.4)	0.331
Headache	7 (22.6)	13 (39.4)	0.183
Itchiness	0 (0)	2 (6.1)	0.493
Shivering	5 (16.1)	1 (3.0)	0.099
Respiratory depression	0 (0)	0 (0)	
Satisfaction score (0/1/2/3)	1/0/10/20	4/0/13/16	0.276

Values are mean ± SD or number of incidence (%). PACU: post-anesthesia care unit. Satisfaction score (0 = un-satisfied, 1 = partial-satisfied, 2 = satisfied, 3 = full- satisfied)

## Abbreviations

ASA: American Society of Anesthesiologists
BIS: bispectral index
KFDA: Korea Food and Drug Administration
MFDS: Ministry of Food and Drug Safety
NRS: numeric rating scale
PACU: post-anesthesia care unit
PCA: patient-controlled analgesia
PONV: postoperative nausea and vomiting
TLH: total laparoscopic hysterectomy
VPS: verbal pain score
